# A Systematic Review of Right Ventricular Diastolic Assessment by 4D Flow CMR

**DOI:** 10.1155/2019/6074984

**Published:** 2019-03-14

**Authors:** Natasha Barker, Benjamin Fidock, Christopher S. Johns, Harjinder Kaur, Gareth Archer, Smitha Rajaram, Catherine Hill, Steven Thomas, Kavitasagary Karunasaagarar, David Capener, Abdullah Al-Mohammad, Alexander Rothman, David G. Kiely, Andrew J. Swift, James M. Wild, Pankaj Garg

**Affiliations:** ^1^Department of Infection, Immunity & Cardiovascular Disease, University of Sheffield, Sheffield, UK; ^2^Sheffield Teaching Hospitals NHS Foundation Trust, Sheffield, UK; ^3^Sheffield Pulmonary Vascular Disease Unit, Royal Hallamshire Hospital, Sheffield, UK

## Abstract

**Background:**

Four-dimensional flow cardiovascular magnetic resonance (4D flow CMR) is a noninvasive novel imaging technology that can be used to visualise and assess right ventricular function. The aim of this systematic review is to summarise the literature available on 4D flow CMR methods used to determine right ventricular diastolic function.

**Methods:**

A systematic review of current literature was carried out to ascertain what is known about right ventricular assessment by quantification of 4D flow CMR. Structured searches were carried out on Medline and EMBASE in December 2018. PG and NB screened the titles and abstracts for relevance.

**Results:**

Of the 20 articles screened, 5 studies met eligibility for systematic review. After a further search on pubmed 1 more relevant article was found and added to the review.

**Conclusions:**

These proposed methods using 4D flow CMR can quantify right ventricular diastolic assessment. The evidence gathered is mainly observational, featuring single-centred studies. Larger, multicentre studies are required to validate the proposed techniques, evaluate reproducibility, and investigate the clinical applicability that 4D flow CMR offers compared to standard practices.* PROSPERO registration number* is CRD42019121492.

## 1. Introduction

Right ventricular diastolic dysfunction (RVDD) is an independent factor contributing to right heart failure. In addition, it is also an independent predictor for nonfatal hospital admissions for heart failure [1]. RVDD is defined by increased right ventricular (RV) filling pressures, caused by passive (RV chamber stiffness) and active (impaired RV relaxation) mechanical abnormalities of the ventricular muscle function during diastole. RVDD was first described by Riggs in 1993 [2]. Since then, it has been shown to be associated with several cardiovascular diseases [3–5]. The prevalence and the incidence of RVDD remain unknown.

Currently, definitive RVDD diagnoses can only be made by invasive right heart catheterisation, to measure RV diastolic pressures. Therefore, novel flow based comprehensive and noninvasive evaluations are needed. A number of noninvasive imaging methods including echocardiographic tissue Doppler assessment have been proposed for RV diastolic assessment. In clinical practice the predominant method for assessment of RV diastolic function is tissue Doppler and investigation of tricuspid inflow. Furthermore, increased RA pressures can be suggestive of RVDD, as well as assessment of size and collapsibility of the inferior caval vein, or determination of flow through the hepatic veins [6]. However, this remains technically challenging due to the complex RV geometry, its motion, and complex three-dimensional tricuspid valve inflow. Even two-dimensional (2D) phase contrast (PC) magnetic resonance imaging (MRI) suffers significant through-plane motion and an accurate assessment of tricuspid inflow is not possible using a static acquisition PC plane. In addition, standard imaging is limited to one directional flow imaging, limiting mechanistic insight into intracavity three-dimensional flow associated with RVDD.

Four-dimensional (4D) flow cardiovascular magnetic resonance (CMR) imaging enables the assessment of intraventricular blood flow in all directions [7, 8]. Several methods of 4D flow quantification have been investigated in cardiovascular diseases including retrospective valve tracking (RVT), energetics, and particle tracing [9]. These 4D flow methods may circumvent the limitations of 2D, unidirectional velocity encoded standard flow imaging. Despite the growing body of 4D flow literature, it remains unclear which method has evidence for RV diastolic assessment. This systematic review comprehensively summarizes these methods of 4D flow CMR that have been applied to the assessment of RV diastolic function. In addition, this review will summarise all the mechanistic insights into RV intracavity blood flow 4D flow CMR.

## 2. Methods

### 2.1. Systematic Review Registration

At inception, this systematic review was prospectively registered (CRD42019121492) with the international prospective register of systematic review (PROSPERO), which is an international database of prospectively registered systematic reviews in health, where there is a health related outcome.

### 2.2. Eligibility Criteria

Eligible studies were those which used 4D flow CMR quantification for the assessment of RV diastolic function. We limited our search to peer-reviewed journals, medicine, and human participants. Studies with fewer than 10 patients or those not published in English were excluded.

### 2.3. Search Strategy

A Literature search was carried out on 27/11/2018 using the Scopus database; this database enables a complete search of both MEDLINE and EMBASE. The search method incorporated the following search terms:* right ventricular kinetic energy* (49 results);* diastolic function* (44,080 results);* four dimensional flow right ventricle *(48). All separate searches were then combined. We identified 20 research studies at this stage.

**Figure 1:**
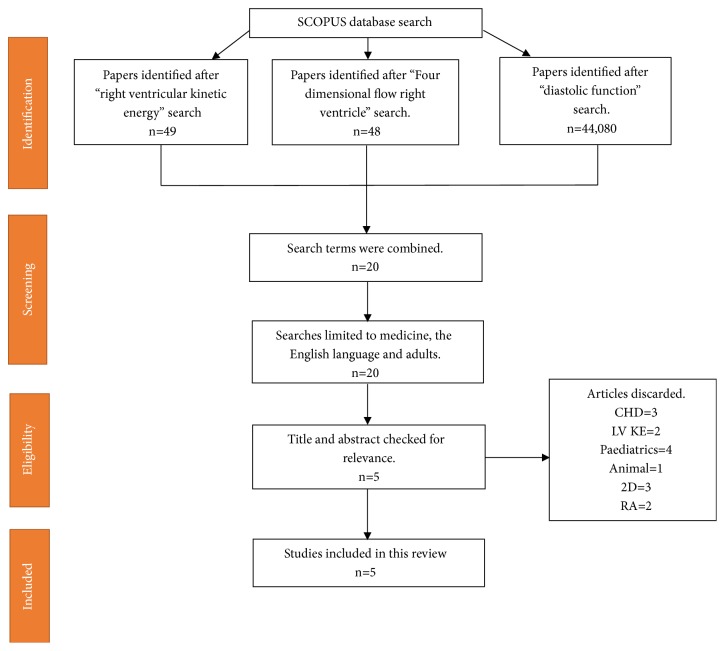
Flow diagram demonstrating evidence synthesis for the systematic review. This flowchart was adapted from Moher et al. using the PRISMA tool [10].

Figure 1 shows literature search flow diagram using the Preferred Reporting Items for Systematic Reviews and Meta-Analyses (PRISMA) tool.

### 2.4. Study Selection

Study selection, review process, and evidence synthesis complied with the PRISMA guidelines [10]. The titles of all the proposed papers were reviewed by PG and the abstracts were reviewed by NB. References quoted in the identified papers were reviewed and no new relevant papers were found.

## 3. Results

After abstract assessment of the 20 research studies, five studies met the inclusion criteria. Research studies were excluded because 3 studies were on congenital heart diseases; 2 studies were on left ventricular function rather than right; 4 studies were in children; 3 more were looking at 2D imaging rather than 4D flow CMR; 2 others involved the atrium rather than the ventricle; and finally, 1 was a preclinical study. A further search was carried out on the pubmed database to make sure any recent publications are not missed (<3 months). One more relevant paper was found. A breakdown of these 6 research papers can be seen in Table 1.

## 4. Discussion

### 4.1. RV Diastolic Functional Assessment

**Figure 2:**
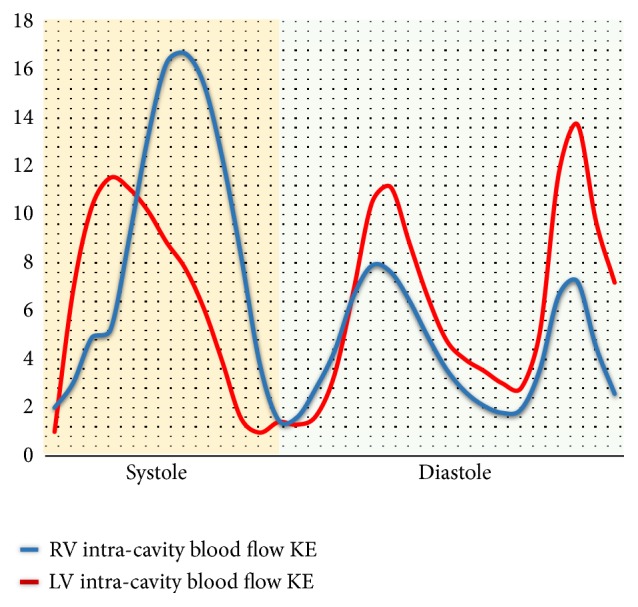
A figure demonstrating quantification of both intraventricular blood flow KE. The RV systolic blood flow KE is much higher than the LV systolic KE. However, the LV early diastolic blood flow KE is higher than RV early diastolic blood flow KE. As other groups have demonstrated, the mean blood flow KE for both the ventricles were comparable (LV: 6.54 uJ/ml versus RV: 6.3uJ/ml).

**Figure 3:**
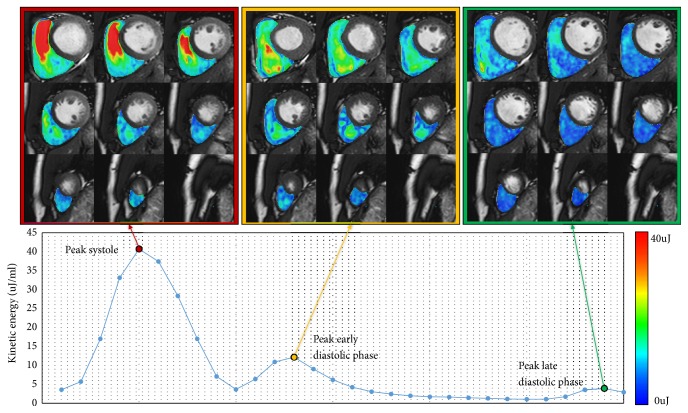
Right ventricular kinetic energy mapping at different phase of the cardiac cycle: red, during peak systole; yellow, during peak early inflow in diastole; green, during peak late inflow in diastole. RV blood flow diastolic KE is lesser than the systolic KE.

4D flow CMR was first used to assess RV diastolic flow in 2011, by calculating the KE of the blood flow.  Figure 2 shows the intraventricular blood flow KE curves for the LV and RV.  Figure 3 shows RV KE during different stages of the cardiac cycle. In 2011 Carlsson et al. calculated the KE of the blood flow during diastole in the left ventricle (LV) and RV to make a comparison [11]. Their results showed that timing and location of blood flow KE differ in each ventricle. There was a significant difference between the LV and RV early diastolic blood flow KEs (6.0±0.6mJ versus 3.6±0.4 mJ;* P*=0.004). However, the average KE of the intracavity blood flow in the left and right ventricles, throughout the whole cardiac cycle, was similar. This suggests that the filling mechanisms during diastole are different in each ventricle. These authors speculated that the difference in filling mechanisms was because of a stronger elastic recoil in the LV due to higher muscle mass causing more suction. It was also speculated that the effect of the atrioventricular (AV) plane movement in the RV had some impact, reducing blood displacement in the RV compared to the LV. Therefore, the blood has a higher velocity as it passes through the mitral valve compared to the tricuspid valve (Figure 3). Further research by Steding Ehrenborg et al., in 2016, has built upon this knowledge and demonstrated that the main determinant of blood flow KE in the RV is RV end-diastolic volume (RVEDV) [12]. This is because the fundamental RV filling mechanism is basal displacement of the AV plane. Investigators of all the studies in this systematic review highlight a need for further investigation of the RV filling mechanism. Additionally, it was found that there was no difference between LV and RV blood flow KE in late diastole (1.1±0.1mJ versus 1.0±0.2mJ, P<0.05) [12], implying that the volume of blood pumped into the ventricles is the same on both sides in late diastole. However, when the end diastolic volume (EDV) and the ejection fraction from each ventricle were calculated, using pathline visualisation, this was not the case. These results showed that the EDV had a tendency to be lower in the RV compared to LV (132±27ml versus 139±25ml, P=0.12) but that the ejection fraction was higher in the RV than the LV (60±5 versus 53±4%* p*<0.01), suggesting that these two factors cancelled each other out and led to no difference in KE between the two ventricles [13].

### 4.2. RV Diastolic Assessment during Exercise

Further RV diastolic blood flow assessment was carried out in two studies looking at 4D flow CMR of participants during exercise, mainly to ascertain whether RV intracavity diastolic blood flow KE changed, what that change was, and how it affected RV work [11, 12]. It was found that during exercise blood flow KE in the RV accounts for 24% of total external RV work compared with 2.8% at rest.

In a case-control study 14 athletes and 14 sedentary healthy adults were recruited and their blood flow was compared during exercise. It was demonstrated that at rest both the sedentary controls and the athletes had a similar RV blood flow KE during diastole. However, at peak exercise the RV diastolic blood flow KE was much higher in the athletes. They proposed that this was caused by enhanced RV diastolic suction due to physiological adaptation seen in the hearts of endurance trained athletes [12].

### 4.3. Flow through the Right Ventricle

**Figure 4:**
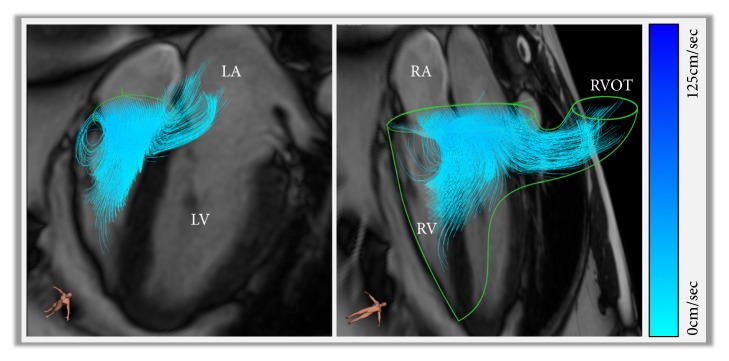
A figure demonstrating complex three-dimensional tricuspid inflow. Panel A: early tricuspid filling and direct flow into the RV outflow tract. Panel B: superimposed view of the anatomy and flow directed into the RVOT early in right ventricular diastole. This figure was made in CAAS software (Pie Medical Imaging, Maastricht, Netherlands).

One area that is relatively unexplored is the functional significance of blood flow through the RV.  Figure 4 demonstrates a visualisation of complex 3D tricuspid inflow. Pathline visualisation was used to assess the direct flow, which is defined as the flow which enters and leaves the ventricle in one cardiac cycle, through the RV. Using the pathline visualisation method, the journey of the blood flow through the RV, during diastole, can be tracked comprehensively. When compared to LV direct flow the location and extension of RV direct flow are different. RV direct flow is located in the basal region of the RV and does not extend as much into the apical region of the ventricle. Additionally, RV direct flow contributed to a larger portion of the EDV than the direct flow of the LV (*P *<0.01), and as stated earlier this is the main determinant of RV intracavity blood flow KE. Furthermore, the distribution of flow through the RV allows a larger capacity of inflow to pass directly to the outflow. Due to this distribution and inflow pattern, there is a preservation of KE during diastole. A high KE at end-diastole will support efficient ejection during systole [13].

Additionally, in a pilot study of patients with primary LV disease, it was found that specific indices of RV flow can detect subtle deterioration in RV function. The harmodynamics in the RV are due to the presence of RV dysfunction. This reduces the amount of direct flow through the ventricle and lowers the KE of the end diastolic blood flow. In comparison these abnormalities would not be picked up on standard CMR or an echocardiogram [14]. Thus, early detection of RVDD could be performed by flow visualisation and energetics calculations from a 4D flow CMR. Also, 4D flow specific measurements could provide novel ways to assess cardiac function as it gives a new pathophysiological insight into interventricular interaction.

The current evidence indicates that, when assessing RV diastolic function, direct flow through the RV is of high importance. This finding also sets a platform for further research evaluating interventricular dependence.

### 4.4. Right Ventricular Diastolic Dysfunction in Disease States

Previous evidence has shown that LV diastolic function is associated with the formation of vortices during diastole [15, 16]. Fredriksson et al. (2011) described how using pathline visualisation of the direct flow through the ventricle forms the vortices [13]. It can also be seen that the vortex rotation velocity slows and broadens as the velocity of the blood flow decreases during diastasis. At end diastole the vortex ring accelerates and becomes more compact. The vorticity of intracavity RV blood flow, which is defined as the localised spinning motion of blood at certain points, was described by Fenster et al. (2015) [17]. In this study the markers of RV diastolic function were significantly different in a control population versus those patients with pulmonary arterial hypertension (PAH). PAH patients had an increased right heart A-wave velocity compared to controls (41±9 versus 26±8,* P*=0.001). PAH patients also had a decreased spatially integrated E-wave velocity (6±1 versus 14±4,* P*<0.001). The overall E/A ratio for the RV was decreased in PAH patients (1.0±0.6 versus 1.8±0.6,* P*=0.012) [17]. They concluded that vorticity can be used as a marker of RV diastolic function, similar to what was proposed by ElBaz et al. for LV diastolic assessment [16]. Further support for this concept was provided in a study by Browning et al., which showed that the differences in 3D right heart flow characteristics between normal and RVDD participants were significant (*p*<0.05) [18]. In this study a comparison of early diastolic right heart (RH) vorticity was made between controls and participants with RVDD. It was found that less total vorticity was displayed in RVDD patients. Additionally, the greatest significant differences were found in the RA. This research provides evidence that vorticity analysis is a viable method for the study of RVDD, and after further work it could be used in clinical investigations.

However, for more specific diagnostic tools to be developed, a greater understanding of the interactions between vorticity, cardiac structure, function, and flow is warranted.

### 4.5. Clinical Implications

Routine assessment of RV diastolic function is not made due to the limitations of current imaging methods. Currently clinicians are unable to quantify subtle changes in RV diastolic function and therefore cannot predict development of right heart failure and adverse RV remodelling by echocardiography. Results from this systematic review have shown that RV diastolic assessment can be made using 4D flow CMR methods and offers mechanistic insight into RVDD. The evidence from this review suggests that RV blood flow energetics can quantify the three-dimensional flow inside the RV throughout the cardiac cycle. RV energetics offer novel insight into diastolic filling patterns and allow the quantification of RV diastolic function. Studies in this systematic review demonstrate that patients with PAH have RVDD, suggesting this may be a novel bioimaging marker in grading the severity of pulmonary hypertension. As this directly measures intracavity flow versus geometric changes which take time to change and remodel, it may become the reference method for RV haemodynamic assessment. In the future, if longitudinal studies demonstrate that it can measure change, RV diastolic assessment by energetics may offer novel therapeutic targets.

### 4.6. Paediatric Applications

The assessment of RV function is important in the clinical management of children with congenital heart disease. In the pediatric population, RV diastolic assessment is even more challenging [18]. Gatzoulis et al. have previously described the importance of adequate assessment of RV diastolic function in paediatric patients that have undergone surgical repair of tetralogy of fallot. They suggested that restriction of RV late diastolic filling, which is indicated by diastolic forward flow in the pulmonary artery after atrial contraction, may reduce pulmonary regurgitation and increase exercise capacity in patients when they are adults [19]. Development of 4D flow CMR techniques could be used to allow accurate serial evaluation in paediatric patients with various congenital heart diseases. This could lead to better prognosis, diagnosis, and treatment.

### 4.7. Reproducibility and Reliability of Proposed Quantification Method

Out of all the 5 studies evaluating RV diastolic function, only two of the studies carried out reproducibility tests. Carlsson et al. in 2011 determined the repeatability of their experiments by performing interscan reproducibility. The KE of the blood flow through the left and RV in the two scanners was very similar. Bias between the two scanners was low (0.58±1.28 mJ) for the RV kinetic energy [11].

Browning et al. performed interobserver reliability tests and found that between a threshold of 0.025s^−1^ and 0.04s^−1^ the concordance coefficient was >0.9 [20].

Although the reliability and reproducibility for 4D flow CMR are not well classified, it has recently been proven to be better than echocardiography in a study by Driessen et al. [21]. They demonstrated that 4D flow CMR was an effective reproducible method to assess TV flow and regurgitation. Inter- and intraobserver quantification produced results with a high correlation (>0.91 and* P*<0.001) to 2D flow across the pulmonary valve (PV), which is the current reference standard [22]. When comparing echocardiography to 4D flow TR quantification methods, 38.5% of patients had different degree of tricuspid regurgitation (TR) by echocardiography. This demonstrates the need for more accurate and reproducible methods.

### 4.8. 4D Flow CMR for Intraventricular Flow Imaging

Some technical adaptations are needed when the focus of flow assessment is mainly intracavity more than valvular flow. This becomes more challenging when assessing stenotic valves as the peak velocity through the valve mandates setting high VENC. For example, in aortic stenosis, this could go up to a VENC of 5m/sec. As already known, this would increase background noise at lower intracavity velocities [23]. Hence, in such cases, a two-VENC approach (low + high) may be needed. This will be at the cost of increased scan time. However, with optimal valvular planning, and whole-heart intracavity flow field-of-view, increase in scan time can be kept to a bare minimum. Even though, for intraventricular flow mapping, a lower VENC is preferred (50-100cm/sec), we recommend using 150cm/sec as this allows quantifying both forward flow and any valvular regurgitation without introducing much background noise for intraventricular velocity mapping [24–27]. For intraventricular flow mapping, different vendors have different validated sequences with significant changes in acceleration methods to achieve shorter scan times [28–30]. Nevertheless, generally, a retrospective gated acquisition is preferred to avoid any temporal blurring in diastole.

### 4.9. Future Work

Three-dimensional, three-directional, time-resolved velocity imaging allows quantification of flow through the RV to assess diastolic function in novel ways. Despite this, there are still some questions left unanswered that require further research. Furthermore, the exact effect of aging on RV diastolic function needs to be determined. Also, the prevalence, incidence, and clinical nature of RVDD need to be investigated in future studies. Additionally, future studies need to evaluate which method of 4D flow quantification is best associated with RVDD and clinical outcomes. Finally, multicentre studies with a larger patient population are needed to investigate the diagnostic utility and further confirm preliminary mechanistic insights from studies included in this systematic review.

### 4.10. Limitations

A systematic review relies on the integrity of the literature that is included. Thus, any bias or limitations of the current literature will affect the reliability of the information provided in this review. The studies covered in this review all have a limited number of participants and most are single-centre studies. Subjectivity when choosing the papers was minimised by the use of two assessing authors.

### 4.11. Conclusion

This systematic review highlights that the current evidence of RV diastolic assessment using 4D flow CMR quantification is mainly based on RV intracavity blood flow energetics. Other 4D flow CMR quantification methods have limited evidence for RV diastolic assessment. RV diastolic dysfunction can be mapped by KE assessment. Further studies evaluating reproducibility, clinical applicability, and advantage of using novel 4D flow derived bioimaging markers of RV diastolic function are warranted. Like with any good scientific report, there are now more questions than answers. However, due to this systematic review, a clearer roadmap has been established.

## Figures and Tables

**Table 1:** Summary of research studies included in the systematic review.

Author(s) (years)	Study Type	n	Method	Type of analysis	Reproducibility	Single Centre
Fredriksson et al (2011)	Mechanistic observational	n=12 HV	Pathline visualisation and kinetic energy	Quantitative	(-)	(+)

Carlsson et al (2011)	Mechanistic observational	n=9 HV	Kinetic energy	Quantitative	(+)	(+)

Steding-Ehrenborg et al (2015)	Mechanistic comparison	n=28 (14 athletes, 14 sedentary controls)	Kinetic energy	Quantitative comparison	(-)	(+)

Fenester et al (2015)	Pilot	n=23 (13 PAH, 10 Controls)	Vorticity And E/A Ratio	Quantitative comparison	(-)	(+)

Fredriksson et al (2016)	Pilot	n=33 (22 Mild IHD, 11 controls)	Pathline visualisation, EDV and ESV	Quantitative	(-)	(+)

Browning et al (2017)	Proof of concept	n=34 (20 with RVDD, 14 controls)	Vorticity And E/A Ratio	Quantitative	(+)	(-)

## Data Availability

The data used to support the findings of this study are included within the article.

## Abbreviations

AV: Atrioventricular
CMR: Cardiovascular magnetic resonance
KE: Kinetic energy
LV: Left ventricle
MRI: Magnetic resonance imaging
PAH: Pulmonary arterial hypertension
PC: Phase contrast
PIV: Particle image velocimetry
RH: Right heart
RV: Right ventricle
RVDD: Right ventricular diastolic dysfunction
RVEDV: Right ventricular end diastolic volume
RVT: Retrospective valve tracking.
